# 1-palmitoyl-2-linoleoyl-3-acetyl-rac-glycerol ameliorates arthritic joints through reducing neutrophil infiltration mediated by IL-6/STAT3 and MIP-2 activation

**DOI:** 10.18632/oncotarget.19384

**Published:** 2017-07-19

**Authors:** Young-Jun Kim, Jae Min Shin, Su-Hyun Shin, Joo Heon Kim, Ki-Young Sohn, Heung-Jae Kim, Jong-Koo Kang, Sun Young Yoon, Jae Wha Kim

**Affiliations:** ^1^ Cell Factory Research Center, Division of Systems Biology and Bioengineering, Korea Research Institute of Bioscience and Biotechnology, Daejeon, Republic of Korea; ^2^ Department of Pathology, EulJi University School of Medicine, Daejeon, Republic of Korea; ^3^ Enzychem Lifesciences, Daejeon, Republic of Korea; ^4^ Department of Laboratory Animal medicine, College of Veterinary medicine, Chungbuk National University, Cheongju, Republic of Korea

**Keywords:** PLAG, collagen-induced arthritis, IL-6, STAT3, neutrophil, Immunology and Microbiology Section, Immune response, Immunity

## Abstract

The pathogenesis of rheumatoid arthritis (RA) has been implicated neutrophil extracellular traps (NETs) formation which could generate autoantigen. Neutrophil contributes to initiate and maintain the inflammatory process in the joint. In this study, we show that 1-palmitoyl-2-linoleoyl-3-acetyl-rac-glycerol (PLAG) decreases neutrophil migration by regulating the activity of STAT3, a regulator of IL-6 and MIP-2 expression. PLAG caused a decrease in IL-6 production in the RAW264.7 macrophage cell line and in rheumatoid arthritis–fibroblast-like synoviocytes via the regulation of STAT3 signaling without affecting NF-κB signaling. In a mouse model of collagen-induced arthritis (CIA), arthritic symptoms were recapitulated, with increased IL-6 level in the synovium, and PLAG treatment restored IL-6 to a level comparable to that achieved with commercial therapeutics (such as Remicade or methotrexate). Staining of joint tissue with neutrophil-specific antibody showed that PLAG significantly reduced the infiltration of neutrophils into the joint synovium of CIA mice. The inhibitory effect of PLAG on IL-6/STAT3 or MIP-2 signaling also reduced the migration of differentiated neutrophils *in vitro*. Therefore, PLAG inhibits the infiltration of destructive neutrophils into inflammatory sites, and can be utilized as a potent therapeutic agent for the treatment of sustained inflammation and joint destruction.

## INTRODUCTION

Arthritis is characterized by joint inflammation, which can be further classified as osteoarthritis, rheumatoid arthritis (RA), or gout. RA is a chronic inflammatory disease that causes progressive destruction of the extracellular matrix of bone and cartilage resulting in irreversible joint damage, deformity, and significant disability. As a soft layer of connective tissue lining the joint cavity, the synovium is a major target of the inflammatory process in RA. Normal synovium contains relatively few types of cells, including macrophage-like synoviocytes (MLSs) and fibroblast-like synoviocytes (FLSs), which are activated to proliferate and invade at the onset of joint inflammation [1, 2]. Synovium becomes hyperplastic and locally invasive at the interface of cartilage and bone, causing progressive destruction mediated by cytokine-induced degradative enzymes, notably the matrix metalloproteinases (reviewed in [3, 4]). A heterogeneous group of inflammatory cells, including lymphocytes, activated macrophages, and plasma cells, are subsequently recruited to the rheumatoid joint along a chemotactic gradient where they are stimulated and contribute to local destruction [5, 6].

Neutrophils contribute to the pathogenesis of many inflammatory diseases. One of the earliest clinical signs of inflammation in an inflammatory arthritis model is the presence of neutrophils in the synovial regions of ankle joints [7]. Ultrastructural studies of cartilage revealed immune complexes embedded in the superficial layers of the synovium [8], thereby providing a solid surface to facilitate neutrophil adherence and activation. Neutrophils play critical roles in initiating and maintaining inflammatory processes in the joint where they accumulate, engulf immune complexes, and release proteolytic enzymes, causing rheumatic tissue destruction [9, 10]. Neutrophil migration is regulated by response to chemokine gradients and IL-8 and MIP-2 are especially the major chemokine of neutrophil migration [11, 12]. RA synovial fluid is primarily characterized by the abundance of major pro-inflammatory cytokines, such as IL-1β and tumor necrosis factor alpha (TNF-α), and chemotactic cytokine, such as IL-8, produced mainly by MLSs, and IL-6, produced by FLSs [3, 13, 14]. Therapeutic antibodies or inhibitors of these inflammatory cytokines exhibit dramatic therapeutic effects in inhibiting the progression of the chronic inflammation [15-18]. In addition to IL-8 or MIP-2, IL-6 functions in the recruitment of neutrophils into the regions of inflammation [19, 20]. IL-6 signals via the heterodimeric IL-6 receptor (IL-6R)/gp130 receptor complex, whose engagement triggers activation of Janus kinases (JAKs) and the downstream effector signal transducer and activator of transcription 3 (STAT3) [21, 22]. STAT3 activation is followed by upregulation of IL-6, which in turn induces STAT3 activation in an autocrine manner [23]. Inhibitors of IL-6R activation or of STAT3 function can be effective in the treatment of inflammatory diseases, including RA. Indeed, tocilizumab, an IL-6-targeting antibody, is among the most effective therapeutic antibodies for the treatment of RA.

A monoacetyldiglyceride, 1-palmitoyl-2-linoleoyl-3-acetyl-rac-glycerol (PLAG) has been isolated from the antlers of Sika deer (*Cervus nippon* Temminck) and chemically synthesized as a single compound with immune-modulatory functions [24-28]. In this study, we demonstrate that PLAG regulates the transcriptional activity of STAT3, which is a key mediator of chronic inflammation and joint destruction in RA, and the consequent blockade of the cytokine amplification loop of IL-6-STAT3 signaling that results in the inhibition of neutrophil migration. In a mouse model of collagen-induced arthritis (CIA), PLAG administration inhibited the progression of RA phenotypes by reducing IL-6 expression and neutrophil infiltration into the arthritic joints, demonstrating its therapeutic efficacy.

## RESULTS

### PLAG decreased immunogen-induced IL-6 and MIP-2 expression

**Figure 1:**
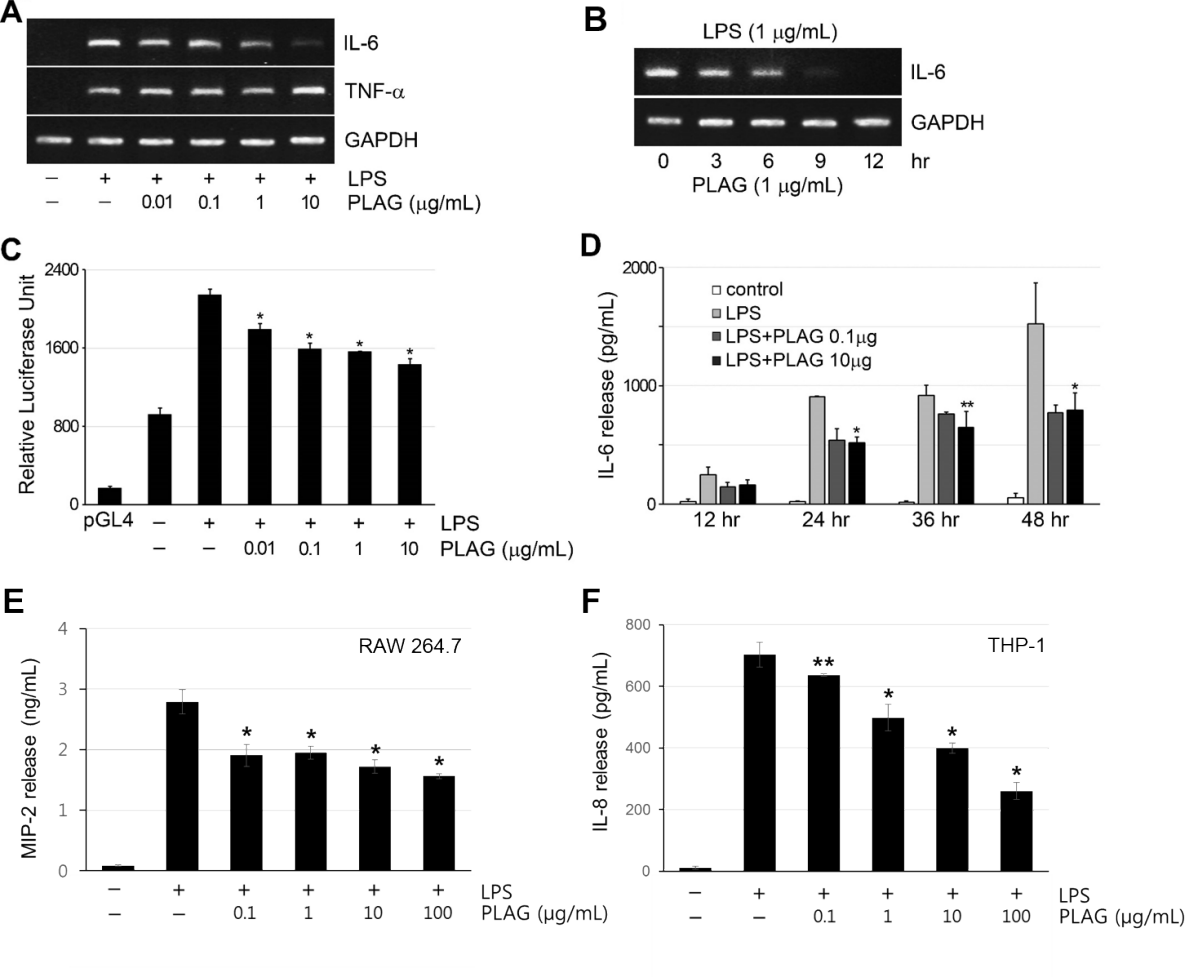
PLAG inhibited IL-6 expression specifically **A.** RAW264.7 cells were treated with LPS (1 μg/mL) for 12 h, to induce expression of inflammatory cytokines IL-6 and TNF-α and co-treated with the indicated concentration of PLAG. Co-treatment with PLAG inhibited LPS-induced IL-6 expression in a dose-dependent manner. However, PLAG did not affect the expression of TNF-α. **B.** LPS-treated RAW264.7 cells were analyzed at the indicated times, showing that PLAG inhibited IL-6 transcription in a time-dependent manner. **C.** Luciferase activity in RAW264.7 cells transfected with pGL4-IL6p was enhanced by treatment with LPS (1 μg/mL). Co-treatment with PLAG inhibited the luciferase activity dose-dependently. *p < 0.005. **D.** IL-6 secreted from LPS-treated RAW264.7 cells was analyzed by ELISA. LPS-induced IL-6 production was inhibited by PLAG (0.1 μg/mL or 10 μg/mL) to a similar level at both concentrations. *p < 0.005, **p < 0.01. **E.**-**F.** MIP-2 secreted from LPS-treated RAW264.7 (E) and THP-1 (F) cells was analyzed by ELISA. LPS-induced MIP-2 secretion was decreased by PLAG in a concentration-dependent manner. *p < 0.005, **p < 0.05.

To test the immune-modulatory activity of PLAG, an inflammatory condition was induced by treating RAW 264.7 macrophages with the immunogen lipopolysaccharide (LPS), and expression of the major pro-inflammatory cytokines IL-6 and TNF-α was analyzed by RT-PCR. The expression of both cytokines was induced by LPS treatment. Unlike TNF-α, whose expression was not affected by PLAG, IL-6 expression was specifically inhibited by PLAG in a concentration- and time-dependent manner (Figure 1A and 1B). IL-6 transcription in RBL-2H3, U937, and NK-YS cells was also affected by PLAG in a similar manner (Supplementary Figure 1A ∼ 1C). Reporter assay using the IL-6 promoter supported the transcriptional regulation of IL-6 expression by PLAG. IL-6 promoter activity was enhanced by LPS, and this was inhibited by co-treatment with PLAG in a concentration-dependent manner (Figure 1C). Secreted IL-6 accumulated with time of LPS treatment, but co-treatment with PLAG kept the IL-6 level at 50-60% of that of the LPS-only-treated control groups (Figure 1D and Supplementary Figure 1D). The specificity of PLAG for IL-6 expression was verified by analyzing the level of secreted TNF-α in the culture medium, which was not affected by PLAG (Supplementary Figure 1E and 1F). Additionally, to determine whether PLAG decreases LPS-induced MIP-2 secretion, the effects on MIP-2 (IL-8) secretion by PLAG were examined in RAW 264.7 and THP-1 cells. Secreted MIP-2 by LPS treatment was significantly decreased by PLAG in a concentration-dependent manner (Figure 1E and 1F). We can conclude that PLAG affects IL-6 and MIP-2 production specifically by regulating its expression.

### PLAG regulated IL-6 expression by modulating STAT3 signaling

**Figure 2:**
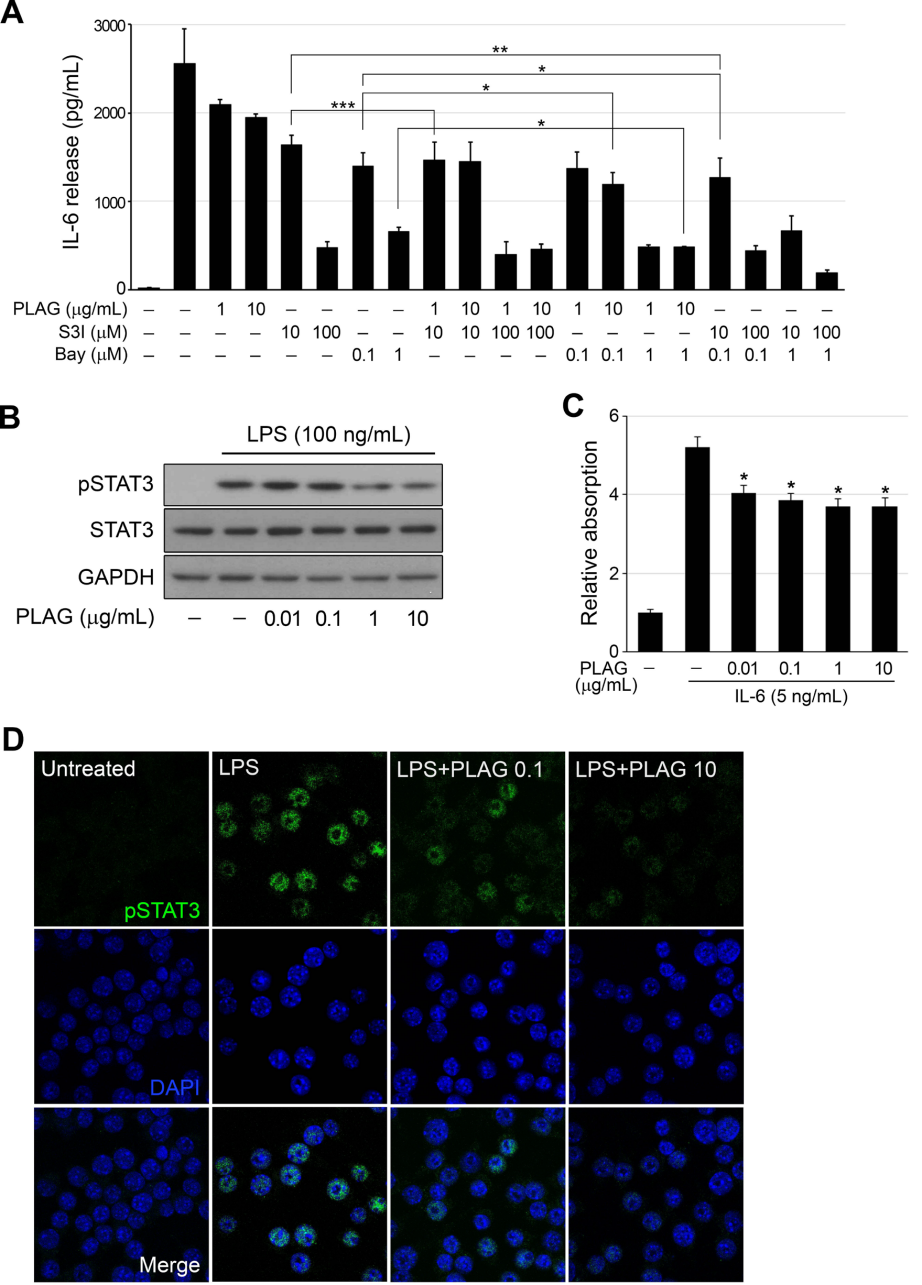
PLAG regulated activation of STAT3 **A.** LPS (100 ng/mL)-treated RAW264.7 cells were treated with Bay 11-7082 (0.1 or 1 μM) or S3I-201 (10 or 100 μM) alone or together with PLAG (1 or 10 μg/mL) for 12 h, and production of IL-6 was analyzed by ELISA of the culture medium. PLAG enhanced the inhibition of IL-6 production by both compounds. *p < 0.005, **p < 0.01, ***p < 0.005. **B.** PLAG inhibited the phosphorylation of STAT3 in LPS-treated RAW264.7 cells in a concentration-dependent manner. **C.** HEK-Blue IL-6 cells were treated with IL-6 and the indicated concentration of PLAG for 24 h. IL-6 induced the expression of SEAP, which is regulated by the STAT3-binding domain, and PLAG inhibited the IL-6 induced STAT3 activation, resulting in a decrease in SEAP production. *p < 0.005. **D.** The activated STAT3 was translocated into the nucleus of LPS-treated RAW264.7 cells in a concentration-dependent manner.

STAT3 and NF-κB, activated by LPS stimulation via the TLR4-mediated pathway, are the major transcription factors regulating IL-6 expression. Co-treatment of cells with PLAG and STAT3 inhibitor (S3I-201) or NF-κB inhibitor (Bay 11-7082) shown an additive effect on IL-6 repression (Figure 2A). Individual treatment of cells with S3I-201 or Bay 11-7082 showed strong repression of LPS-induced IL-6 production, which was slightly enhanced by PLAG co-treatment in a dose-dependent manner. Both inhibitors also exhibited synergy in repression of IL-6 expression. Western blot analysis of LPS-stimulated RAW 264.7 cells showed that PLAG attenuated phosphorylation of STAT3 (Figure 2B). The regulation of STAT3 signaling by PLAG was verified in HEK-Blue IL-6 cells, which carry the IL-6-inducible secreted embryonic alkaline phosphatase (SEAP) gene regulated by STAT3 responsive element. IL-6-induced SEAP activity in HEK-Blue IL-6 cells was inhibited by the addition of PLAG (Figure 2C). Nuclear localization of activated STAT3 in LPS-stimulated RAW 264.7 cells was decreased by PLAG in a dose-dependent manner (Figure 2D).

### Arthritic phenotypes in mouse model of CIA were alleviated by PLAG

**Figure 3:**
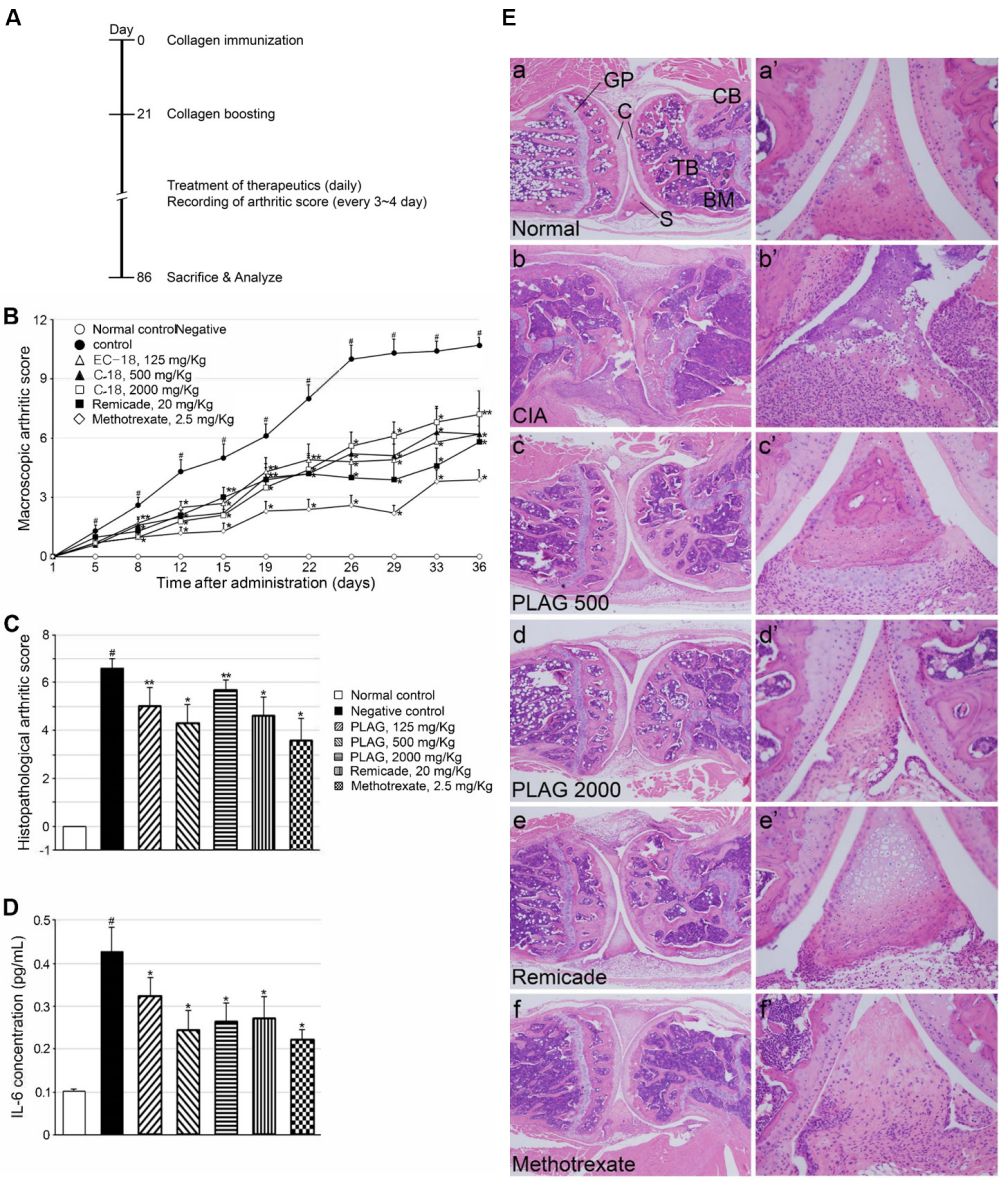
Pathologic phenotypes of CIA mice were ameliorated by PLAG **A.** Scheme for generation of CIA mouse model. **B.** RA was induced by injection of collagen in DBA/1J mice, and mice were examined visually during development of RA and scored based on the severity of their phenotypes. The arthritic phenotypes in CIA mice were ameliorated by PLAG treatment. Ten mice in each group were examined and mean values determined. Values represent mean ± standard error of the mean (SEM). **C.** Blood was collected from each mouse, and IL-6 levels in plasma were analyzed by ELISA. The increased IL-6 level in the blood was decreased by the administration of PLAG in CIA mice. **D.** The level of antibodies against type II collagen was not affected by PLAG. Each sample was analyzed in triplicate, and values represent mean ± SEM. #*p* < 0.01 (Control vs. CIA, Aspin-Welch *t*-test), *p < 0.01, **p < 0.05 (CIA vs. treatment, Dunnett’s *t*-test). E. The articular joints from the rear legs were analyzed by H&E staining. The damage to the cartilage, synovium, and bone marrow observed in CIA mice was alleviated by the administration of PLAG (500 or 2000 mg/kg), Remicade (20 mg/kg), or methotrexate (2.5 mg/kg). a-f. Representative images of the CIA joint are shown. A population of infiltrated immune cells are positively stained in the synovium of a CIA mouse (b’). Images were taken at 40× (a-f) and 200× (a’-f’) magnification. C, cartilage; S, synovium; TB, trabecular bone; CB, compact bone; BM, bone marrow; GP, growth plate.

*In vivo* efficacy of the inhibitory effect of PLAG on IL-6 expression was studied in a mouse model of arthritis (Figure 3A). Oral administration of PLAG alleviated arthritic phenotypes induced by collagen injection. Erythema and swelling of the ankles and joints of CIA mice were markedly alleviated by the administration of PLAG, to a level similar to that achieved with commercial RA therapeutics such as Remicade and methotrexate (Figure 3B). The serum level of IL-6 in CIA mice was significantly decreased by the administration of PLAG or commercial therapeutics, but not by antibody against the injected collagen (Figure 3C and 3D), suggesting that production of the inflammatory cytokine is correlated with the severity of arthritic phenotypes. H&E tissue staining showed the pathological phenotypes of the lesions, such as the swelling of synovium, destruction of collagen layers, infiltration of bone marrow and blood, and deformity of the joints, which were alleviated by PLAG in a concentration-dependent manner (Figure 3E). Representative images of stained tissue are shown in Figure 3E.

### Neutrophil infiltration into the joints was decreased by PLAG

**Figure 4:**
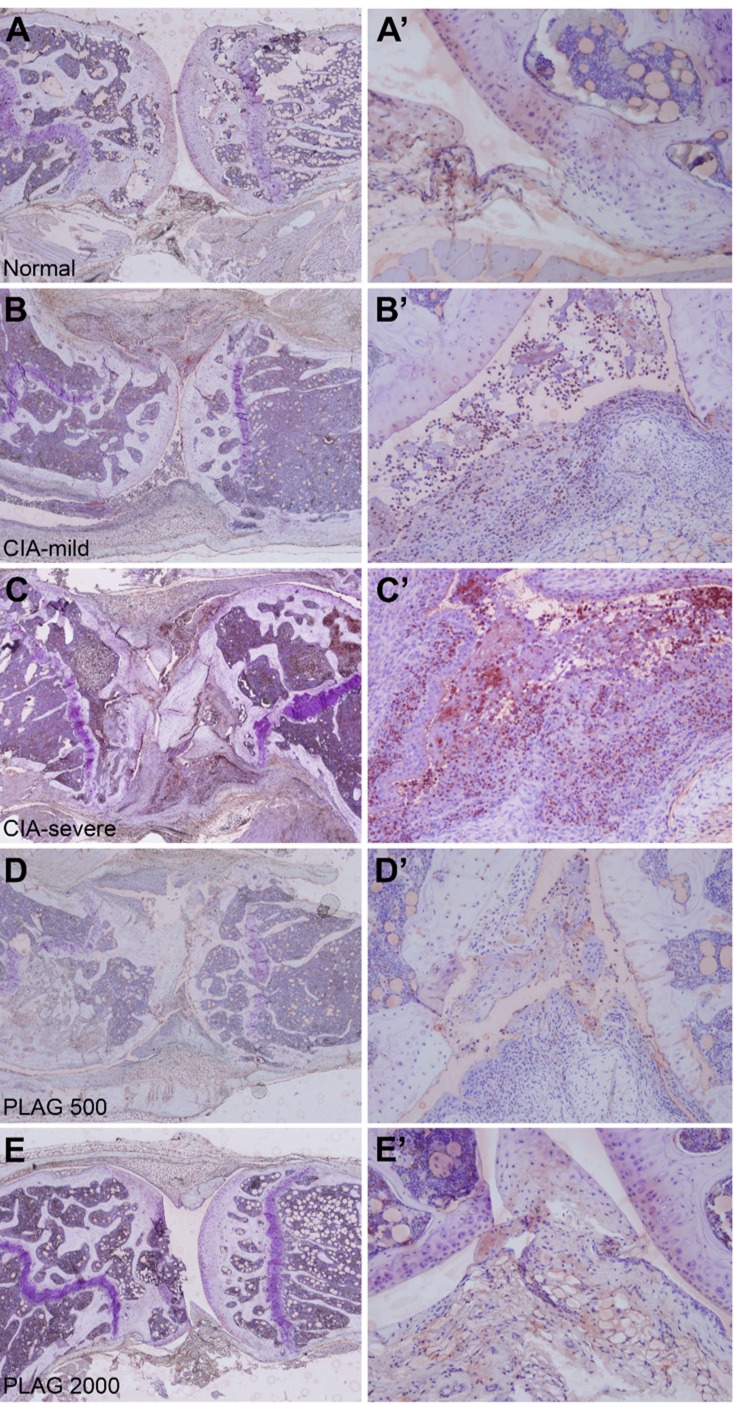
PLAG inhibited neutrophil infiltration into CIA joints **A.**-**E.**, A’-E’. Articular joints of rear legs from control, untreated CIA, and treated CIA mice were stained with a neutrophil-specific antibody (NIMP-R14). Images were taken at 40× (A-E), and 200× (A’-E’, B”-C”) magnification. Neutrophil migration into the CIA joints was correlated with the severity of RA (B-C”). The number of infiltrated neutrophils was significantly decreased by PLAG treatment (D-E”).

**Figure 5:**
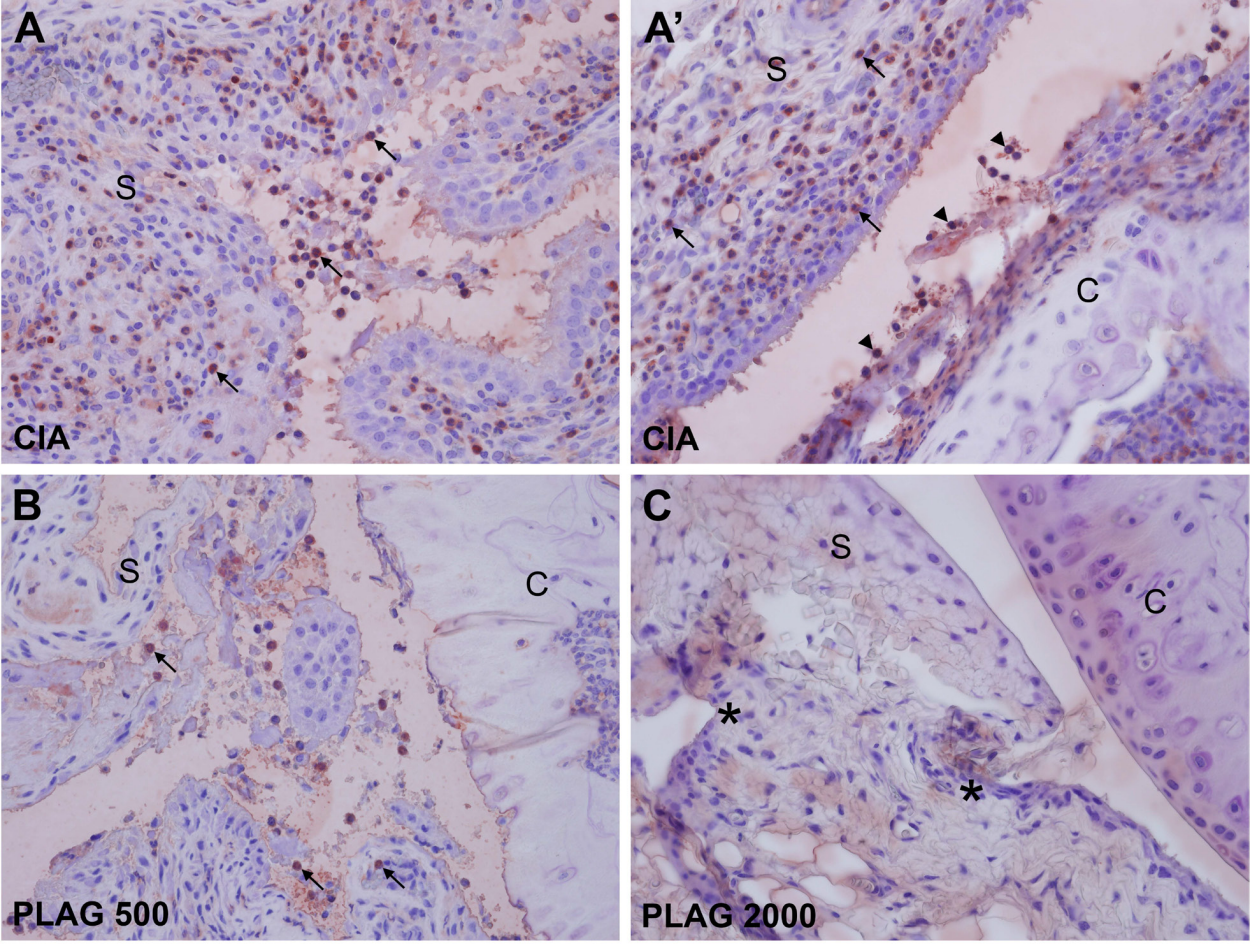
PLAG inhibited neutrophil infiltration into the arthritic joint **A.**-A’. Large numbers of neutrophils were detected in the articular joints. The synovial region (A) and the area between the synovium and cartilage (A’) are shown at 400× magnification. Some of the infiltrated neutrophils are marked by an arrow (in the synovium) or arrowhead (at the surface of cartilage). **B.**-**C.** Neutrophil infiltration was significantly decreased by the administration of PLAG. A small number of neutrophils was detected in the synovium of low-dose-PLAG-treated joint (B, arrow). Neutrophils were barely detectable in the joint of high-dose-PLAG-treated joint, although scars (*) from the previously damaged tissues remained (C).

As a pro-inflammatory chemokine, IL-6 and MIP-2 regulates immune cell migration. Based on H&E staining shown in Figure 4 and 5, many immune cells were detected in the joints of CIA mice and infiltrated the synovium. Interestingly, the population of neutrophils that had migrated into the joint was increased in the CIA mice (Figure 4B’ and 4C’). In wild-type mice, no neutrophils were detected in the articular joint regions (Figure 4A and A’). However, neutrophil numbers in the synovium and collagen surfaces were markedly increased in CIA mice (Figure 4C and 4C’), which correlated with arthritic severity (Figure 5A and 5A’). The increased number of infiltrated neutrophils in the rheumatoid joint was decreased by the administration of PLAG in a dose-dependent manner (Figure 4D, 4E, 5B, and 5C).

**Figure 6:**
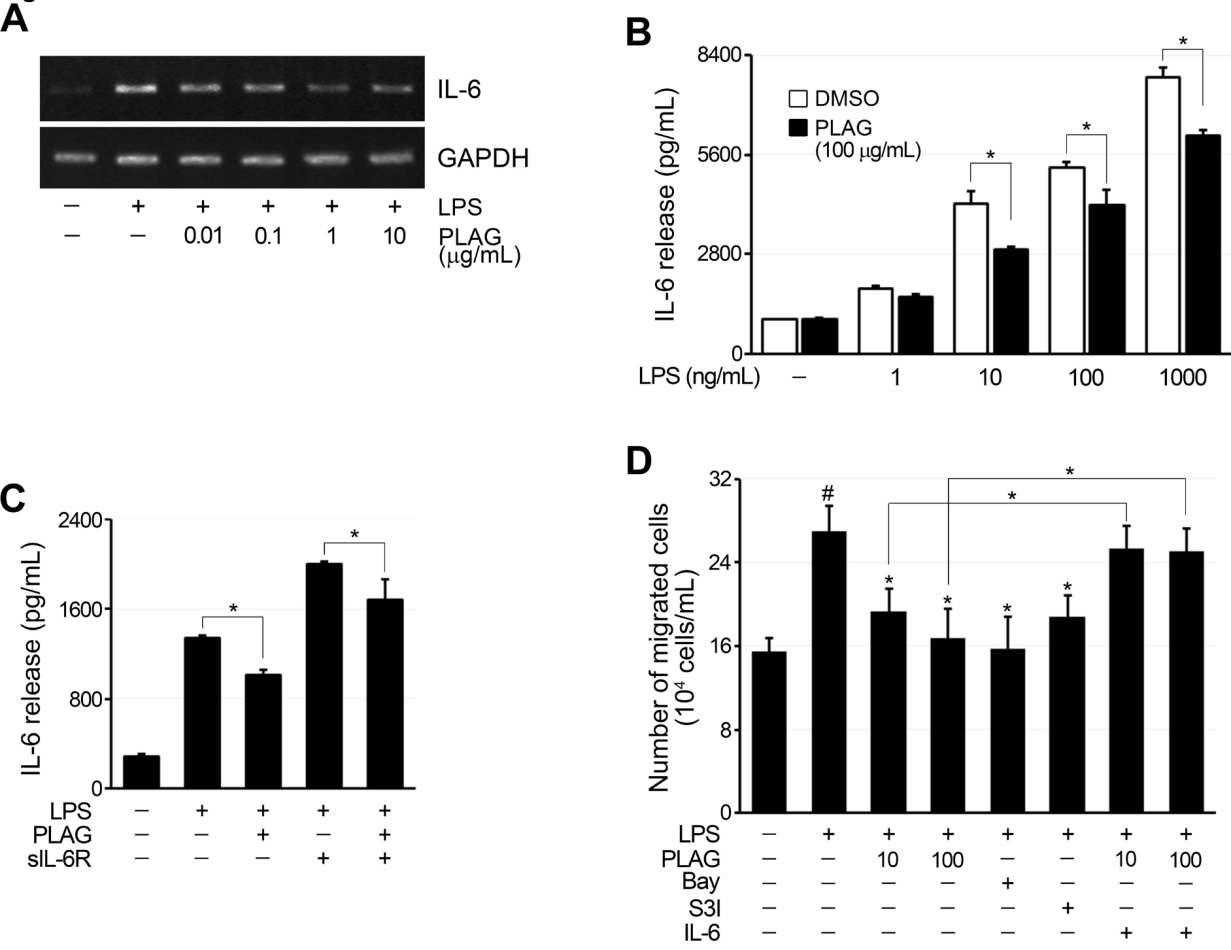
The migration of neutrophils was inhibited by PLAG **A.** LPS treatment (100 ng/mL) of RA-FLSs increased IL-6 expression, which was inhibited by co-treatment with PLAG in a concentration-dependent manner. **B.** IL-6 production by LPS-stimulated RA-FLSs was dependent on LPS concentration, and the inhibitory effect of PLAG was significant only after IL-6 production reached a certain level. *p < 0.005. **C.** The addition of sIL-6R to LPS-stimulated RA-FLSs further increased IL-6 expression by the cells, and this was also blocked by co-treatment with PLAG. *p < 0.005, **p < 0.01. D. Culture medium from LPS-treated RA-FLSs induced the migration of differentiated HL-60 cells. The migration of differentiated HL-60 cells was significantly inhibited by PLAG, and this was restored by the addition of recombinant IL-6 to the culture medium of PLAG-treated cells.

To verify the relationship of IL-6 production by synoviocytes with neutrophil chemotaxis, an *in vitro* assay system was established using FLSs from RA patients. The expression of IL-6 in RA-FLSs was increased by LPS treatment, and this was inhibited by PLAG (Figure 6A and B). Secreted IL-6 stimulates IL-6 receptor to amplify IL-6 production via the STAT3 pathway. IL-6 production in LPS-stimulated RA-FLSs, which do not express IL-6R, was amplified by co-treatment with soluble IL-6R (Figure 6C). This increase in IL-6 production in RA-FLSs was also inhibited by PLAG (Figure 6C). Chemotaxis of differentiated HL-60 (dHL-60) cells toward the RA-FLS culture medium was induced by treating RA-FLSs with LPS and inhibited by co-treatment with PLAG or inhibitors of NF-κB and STAT3 (Figure 6D). Noticeably, the reduced chemotaxis of dHL-60 cells was recovered by addition of IL-6 to the culture medium of PLAG-treated RA-FLSs, demonstrating that PLAG inhibits dHL-60 cell chemotaxis by reducing the production of IL-6 by synoviocytes.

## DISCUSSION

As major phagocytes in the circulation, neutrophils are the first line of defense in the early phase of innate immune responses to infections [29-31]. However, neutrophils can cause tissue damage because of their activities, such as secretion of tissue-degrading enzymes, unless controlled appropriately. Accumulation of neutrophils in the joint synovium causes the inflammation to become chronic and contribute to the progression of RA [32-34]. In this study, the efficacy of PLAG in the suppression of IL-6 and MIP-2 (IL-8 or CXCL8) expression, which stimulates neutrophil migration, and its therapeutic effect on RA treatment were demonstrated (Figure 1 and 2).

RA is a complex disease resulting from both defined and undefined causes, but it is characterized by the presence of pro-inflammatory cytokines, including IL-6 and MIP-2. The concentration of IL-6 increases both in the joint synovium and the blood of RA patients [35, 36], and this was recapitulated in the mouse model of CIA (Figure 3). PLAG administration specifically inhibited IL-6 production to a level similar to that achieved with Remicade or methotrexate (Figure 3D). The serum level of anti-collagen antibodies was also increased in the arthritis model. However, the increased level of anti-collagen antibodies was not changed by treatment with any therapeutic, including PLAG. As a pro-inflammatory cytokine, IL-6 participates in the perpetuation of synovial inflammation resulting in joint destruction by stimulating neutrophil migration, osteoclast maturation, and vascular endothelial growth factor (VEGF)-stimulated pannus proliferation [37]. MIP-2 is one of the major neutrophil chemoattractant factors [38, 39]. MIP-2 levels has been reported to be elevated in an experimental arthritis model [40], and neutralization of MIP-2 has been found to significantly decrease the severity of CIA [41]. In this study, the effects of PLAG on the regulation of neutrophil infiltration into the joint synovium was demonstrated in a mouse CIA model. We have been found that PLAG decreased the MIP-2 secretion in macrophage cells (Figure 1E and 1F). The inhibition of neutrophil migration by PLAG was partially recovered by addition of IL-6 in the culture medium, demonstrating that PLAG modulates neutrophil infiltration into the joint by regulating IL-6 expression in synoviocytes (Figure 6). In addition, the deformation of joint synovium by proliferating pannus was eliminated by PLAG treatment, and expression of receptor activator of NF-κB ligand (RANKL) in RA-FLSs was also inhibited by PLAG, suggesting that PLAG might also be effective in the regulation of osteoclast development (data not shown). Collectively, these data suggest that the inhibitory effect of PLAG on IL-6 and MIP-2 expression in joint synoviocytes may be used to prevent arthritis development and joint destruction.

Although STAT3 can also be activated by LPS, NF-κB is the major target of LPS stimulation, which induces the expression of both pro-inflammatory cytokines, TNF-α, IL-6, and MIP-2 in macrophages . However, the inhibitory effect of PLAG was shown to be selective for IL-6 and MIP-2 (Figure 1). Unlike TNF-α, whose expression is primarily regulated by NF-κB, IL-6 and MIP-2 expression can be regulated by the transcriptional activities of both NF-κB and STAT3. PLAG seems to suppress STAT3 signaling pathway but not NF-kB (Supplementary Figure 2). The IL-6 and MIP-2 produced enhance and prolong the activity of STAT3 leading to excessive neutrophil recruitment into the inflamed region, which induces PLAG to reduce the overactivated STAT3 to the normal level. This suppressing function of PLAG on STAT3 activity without any side effects can be beneficial for the treatment of chronic inflammatory diseases, including RA. However, the threshold level of STAT3 activity needed to provoke the inhibitory effect of PLAG must be determined.

In conclusion, the inhibitory effect of PLAG on IL-6, MIP-2, and STAT3 signaling also reduced the migration of differentiated neutrophils *in vitro*. Therefore, PLAG inhibits the infiltration of destructive neutrophils into inflammatory sites, and can be utilized as a potent therapeutic agent for the treatment of sustained inflammation and joint destruction.

## MATERIALS AND METHODS

### Cell culture and reagents

RAW264.7, THP-1, RBL-2H3, U937, and NK-YS cell lines were purchased from American Type Cell Culture (ATCC, Manassas, VA, USA), and maintained in Dulbecco’s modified Eagle medium (DMEM) or RPMI (Welgene, Gyeongsangbuk-do, Republic of Korea) supplemented with 10% heat-inactivated fetal bovine serum (FBS, Welgene) and 100 U/mL penicillin-streptomycin (Thermo Fisher Scientific, Waltham, MA, USA). HL-60 cells were purchased from ATCC and maintained in Iscove’s modified Dulbecco’s medium (IMDM) (Welgene) supplemented with 20% FBS and 100 U/mL penicillin-streptomycin. RA-FLSs were isolated from synovial membranes removed from the joints of RA patients (Soonchunhyang University Hospital, Republic of Korea) and cultured in DMEM supplemented with 20% FBS, 100 U/mL penicillin-streptomycin, and Mycoplasma Elimination Cocktail (1:10,000, Applied Biological Materials, Richmond, BC, Canada). RA-FLSs were used in experiments within eight passages. All cells were maintained at 37°C in a humidified atmosphere of 5% CO_2_. PLAG was obtained from Enzychem Lifesciences (Seoul, Republic of Korea). LPS and Bay 11-7082 were purchased from Sigma-Aldrich (St. Louis, MO, USA), and S3I-201 was from Santa Cruz Biotechnology (Santa Cruz Biotechnology, CA, USA). Recombinant mouse or human IL-6 was purchased from PeproTech (Rocky Hill, NJ, USA).

### Reverse transcription-polymerase chain reaction (RT-PCR)

Total RNA was isolated from cells using TRI Reagent^®^ (MRC, Cincinnati, OH, USA) per the manufacturer’s instructions. One microgram of total RNA was reverse-transcribed using oligo-dT primer, dNTP mix, RNase inhibitor, and Thermo RTase (all from BioAssay, Daejeon, Republic of Korea) for cDNA synthesis, followed by conventional PCR with 2× Taq Premix (BioAssay). The following primers were used in this study: IL-6, 5’-GATGCTACCAAACTGGATATAATC-3’ (forward) and 5’-GGTCCTTAGCCACTCCTTCTGTG-3’ (reverse); TNF-α, 5’-ATGAGCACAGAAAGCATCCGC-3’ (forward) and 5’-CCAAAGTAGACCTGCCCGGACTC-3’ (reverse); IFN-γ, 5’-TGGCTGAACTGTCGCCAGCA-3’ (forward) and 5’-TGGCTGCCTAGTTGGCCCCT-3’ (reverse); and GAPDH, 5’-CCATCACCATCTTCCAGGAG-3’ (forward) and 5’-ACAGTCTTCTGGGTGGCAGT-3’ (reverse).

### Enzyme-linked immunosorbent assay (ELISA)

Cell supernatants were collected and the levels of mouse or human IL-6, MIP-2 (IL-8) and human TNF-α were analyzed using the OptEIA kit (BD Biosciences, San Jose, CA, USA) per the manufacturer’s instructions. TMB One Component HRP Microwell Substrate (Surmodics, Eden Prairie, MN, USA) was used for color development, and optical densities were measured at 450 nm using an Emax Microplate Reader (Molecular Devices, Sunnyvale, CA, USA). Concentrations were calculated from a standard curve generated by a curve-fitting program. Each sample was analyzed in triplicate, and concentrations are presented as mean ± standard deviation (SD).

### Reporter assay

The IL-6 promoter sequence was amplified by PCR from the genomic DNA of RAW264.7 cells using primer pair 5’-ctcgagcaggtgaagaaagtggca-3’ (forward) and 5’-aagctttcctatatttattgggggttgag-3’ (reverse). The PCR product was then inserted into the *Xho*I/*Hin*dIII site of vector pGL4 (Promega, Madison, WI, USA) to generate reporter plasmid pGL4-IL6p. Transfection was performed using Attractene Transfection Reagent (Qiagen, Hilden, Germany) per the manufacturer’s instructions. Briefly, RAW264.7 cells (1×10^5^/mL) were seeded in 48-well plates and cultured overnight followed by transfection with pGL4-IL6p or empty vector (0.5-1 μg/well). After 18 h, cells were treated with 0.01, 0.1, 1 or 10 mg/mL PLAG and 10 ng/mL LPS for 24 h. Transient expression of the reporter gene was quantified using the Dual-Glo^®^ Luciferase Assay System (Promega) per the manufacturer’s instructions. Luminescence was measured on a TD-20/20 Turner Luminometer (Promega). Each sample was analyzed in triplicate, and mean values were compared with control and presented as mean ± SD.

### Secreted alkaline phosphatase (SEAP) assay

HEK-Blue IL-6 cells were obtained from InvivoGen (San Diego, CA, USA) and maintained in DMEM supplemented with 10% FBS and 100 U/mL penicillin-streptomycin. Cells (2.5×10^5^/mL) were plated in 48-well plates and treated with IL-6 (5 ng/mL) and/or various concentrations of 0.01, 0.1, 1, or 10 mg/mL PLAG for 24 h. Twenty microliters of culture medium were mixed with 180 μL Quanti-Blue (InvivoGen), and incubated at 37°C for 1 h. Absorbance at 650 nm was measured using an Emax Microplate Reader. Each sample was analyzed in triplicate, and mean values were compared with control and presented as mean ± SD.

### Western blotting

Cells were lysed in RIPA buffer containing 50 mM Tris-HCl (pH 7.4), 150 mM NaCl, 2 mM EDTA, 1% NP-40, 0.5% sodium deoxycholate, 0.1% SDS, protease inhibitor cocktail (Roche, Indianapolis, IN, USA) and Halt™ phosphatase inhibitor (Thermo Fisher Scientific). The lysates were incubated on ice for 20 min and collected by centrifugation at 13,000 rpm for 20 min at 4°C. The supernatants were removed to clean tubes, mixed with 5× sample buffer, and heated at 100°C for 5 min. Proteins were separated by SDS-PAGE through 10% gels and transferred onto polyvinylidene fluoride (PVDF) membranes (Millipore, Billerica, MA, USA). Membranes were blocked in phosphate-buffered saline (PBS) containing 5% (w/v) bovine serum albumin (BSA, Bioworld, Dublin, OH, USA) and 0.05% (v/v) Tween 20 (PBST) for 1 h and incubated overnight at 4°C with primary antibody against STAT3 or phospho-STAT3 (Cell Signaling Technology, Danvers, MA, USA). Antibody against GAPDH (sc-25778, Santa Cruz Biotechnology) was used as internal control. Membranes were incubated with horseradish peroxidase (HRP)-labeled secondary antibodies (SC-2005, Santa Cruz Biotechnology) for 1 h and visualized using Immobilon Western Chemiluminescent HRP Substrate (Millipore).

### Confocal microscopy

RAW264.7 (1×10^5^/mL) or RA-FLS (1×10^4^/mL) cells were seeded on glass cover slips, cultured overnight, and treated with 0.1 or 10 mg/mL PLAG and LPS for 12 h. Cells were fixed for 20 min in 4% paraformaldehyde in PBS, permeabilized in methanol for 10 min, blocked in 1% BSA, and incubated with anti-phospho-STAT3 (Cell Signalling Technology) or anti-p65 (Santa Cruz Biotechnology) at 4°C overnight. Alexa Fluor^®^ 488- or 594-conjugated donkey anti-rabbit or anti-mouse IgG (Thermo Fisher Scientific) was used as secondary antibody at 1:1000 dilution. Cover slips were washed, dried, and mounted in Prolong Gold Antifade Reagent with DAPI) Thermo Fisher Scientific). Confocal images were acquired using a Zeiss LSM 510 META Laser Scanning Microscope equipped with ZEN software (Carl Zeiss AG, Jena, Germany). Images were processed and analyzed using ImageJ (National Institutes of Health, Bethesda, MD, USA). Representative images are shown.

### Invasion assay

RA-FLS cells (1×10^5^/mL) were seeded in a 24-well plate and cultured overnight. Cells were treated with LPS together with 10 or 100mg/mL PLAG, Bay11-7082, or S3I-201 for 24 h. The culture supernatants were collected and transferred to a 24-well plate, which served as the bottom chamber of a modified Boyden chamber. HL-60 cells were induced to differentiate by treatment with 1.3% dimethyl sulfoxide (DMSO) and 5 μM sodium butyrate (Sigma-Aldrich) for five days. Differentiation of HL-60 cells was verified by analysis of expression of neutrophil-specific genes using RT-PCR, and chemotaxis of the differentiated cells was analyzed using Transwell^®^ Permeable Support (Corning, Corning, NY, USA). The differentiated cells (5×10^4^/mL) were seeded onto polyester membrane inserts with 8.0-μm pores, which served as upper chambers, and inserted into the 24-well plate filled with RA-FLS culture medium. The plate was incubated at 37°C for 16 h, and cells that had migrated from the upper chamber into the lower chamber were counted following Trypan blue staining. Cells in each well were counted three times and the data presented as mean ± SD.

### Animals and CIA

Animal experiments were carried out at Biotoxtech (Chungcheongbuk-do, Republic of Korea). Specific-pathogen-free male DBA/1J mice were housed in a specific-pathogen-free facility under consistent temperature and 12h light/dark cycles. All experimental procedures were approved by the Institutional Animal Care and Use Committee of Biotoxtech and performed in compliance with the National Institutes of Health Guidelines for the care and use of laboratory animals and Korean national laws for animal welfare.

Arthritic mice were generated by collagen treatment as described in Brand et al [42]. Male DBA/1J mice (6-8 weeks of age) were immunized intradermally with 100 µg bovine type II collagen (Sigma-Aldrich) in 25 µL 50 mM acetic acid and emulsified with an equal volume of complete Freund’s adjuvant (CFA, Sigma-Aldrich). On day 21, the animals were received the same treatment, with the exception that incomplete Freund’s adjuvant (IFA, Sigma-Aldrich) replaced CFA. Arthritic animals were then divided into six groups of 10 mice each. PLAG (125, 500, 2000 mg/kg) or methotrexate (2.5 mg/kg) was administered orally each day or Remicade (20 mg/kg) was injected intraperitoneally from the day of boosting for 5 weeks. Olive oil was administered as negative control. Arthritis scores were recorded every 3-4 days. A scoring system was implemented to quantify the objective evaluation of arthritic severity. Knees, ankles, and paws were analyzed macroscopically and rated individually for degree of inflammation on a scale of 0-4. The rating system for severity of inflammation was defined as follows: 0 (no evidence of erythema or swelling); 1 (erythema and mild swelling confined to the tarsals or ankle joint); 2 (erythema and mild swelling extending from the ankle to the tarsals); 3 (erythema and moderate swelling extending from the ankle to metatarsal joints); 4 (erythema and severe swelling encompass the ankle, foot and digits, or ankylosis of the limb). At the end of this assessment, the animals were euthanized in a CO_2_ chamber. Joints were collected for pathological analysis and blood was collected and plasma prepared for determination of IL-6 concentration. Animals that showed no inflammatory signs at 33 days after immunization were excluded. In the present study, 80% of animals immunized with collagen developed arthritis and were included in the CIA group.

### Pathological analysis

Knee joints were removed from the rear legs of control and CIA mice, untreated and treated with PLAG. Immediately after collection, knee joints were fixed in ice-cold 10% formalin, embedded in paraffin, and 4-μm sections were prepared. Each section was deparaffinized after being mounted on a charged glass slide and stained with hematoxylin and eosin (H&E) and examined by light microscopy (Olympus, Tokyo, Japan). Each slide was examined microscopically, and histopathological arthritis indices were prepared using the rating system defined as follows: 0 (no detectable pathology); 1 (hyperplasia of the synovial membrane and presence of polymorphonuclear infiltrates); 2 (pannus and fibrous tissue formation and focal subchondral bone erosion); 3 (articular cartilage destruction and bone erosion); 4 (extensive articular cartilage destruction and bone erosion).

For immunohistochemical staining, the deparaffinized tissues were incubated with 3% hydrogen peroxide in methanol to quench endogenous peroxidase activity, followed by blocking in 1% BSA. Sections were incubated with NIMP-R14 (1:100, Thermo Fisher Scientific) at 4°C overnight. The slides were incubated with HRP-conjugated goat anti-rat IgG (1:250, Santa Cruz Biotechnology) at room temperature for 15 min followed by visualization with 3-amino-9-ethylcarbazole (AEC) substrate (Dako, Glostrup, Denmark). The tissues were counterstained with 10% Mayer’s hematoxylin, dehydrated, and mounted in Crystal Mount (Sigma-Aldrich). An irrelevant goat IgG of the same isotype and antibody dilution solution served as a negative control. Images were obtained under a light microscope (Olympus).

### Statistical analysis

The significance of differences between experimental groups was analyzed using Student’s unpaired *t*-test for *in vitro* analysis or Aspin-Welch or Dunnett’s *t*-test for pathological analysis. Differences in means were considered significant if *p* < 0.05 by comparing cells treated with LPS or IL-6 only with cells co-treated with PLAG. Values represent mean ± SD.

## SUPPLEMENTARY MATERIALS FIGURES



## Abbreviations

PLAG: 1-palmitoyl-2-linoleoyl-3-acetyl-rac-glycerol
NETs: neutrophil extracellular traps
STAT3: signal transducer and activator of transcription 3
RA: rheumatoid arthritis
FLS: fibroblast-like synoviocytes
MLS: macrophage-like synoviocytes
CIA: collagen-induced arthritis
TNF-α: tumor necrosis factor alpha
IL-6R: IL-6 receptor
JAKs: Janus kinases
LPS: lipopolysaccharide
SEAP: secreted embryonic alkaline phosphatase
