# Supplementary material for: 1-palmitoyl-2-linoleoyl-3-acetyl-rac-glycerol ameliorates arthritic joints through reducing neutrophil infiltration mediated by IL-6/STAT3 and MIP-2 activation
Source: Oncotarget. 2017 Jul 19;8(57):96636–48. doi: 10.18632/oncotarget.19384 (PMC5722510; doi:10.18632/oncotarget.19384)
Supplement: Supplementary file 1 [file oncotarget-08-96636-s001.pdf]

1-palmitoyl-2-linoleoyl-3-acetyl-rac-glycerol ameliorates arthritic joints through reducing neutrophil infiltration mediated by IL-6/STAT3 and MIP-2 activation

SUPPLEMENTARY MATERIALS

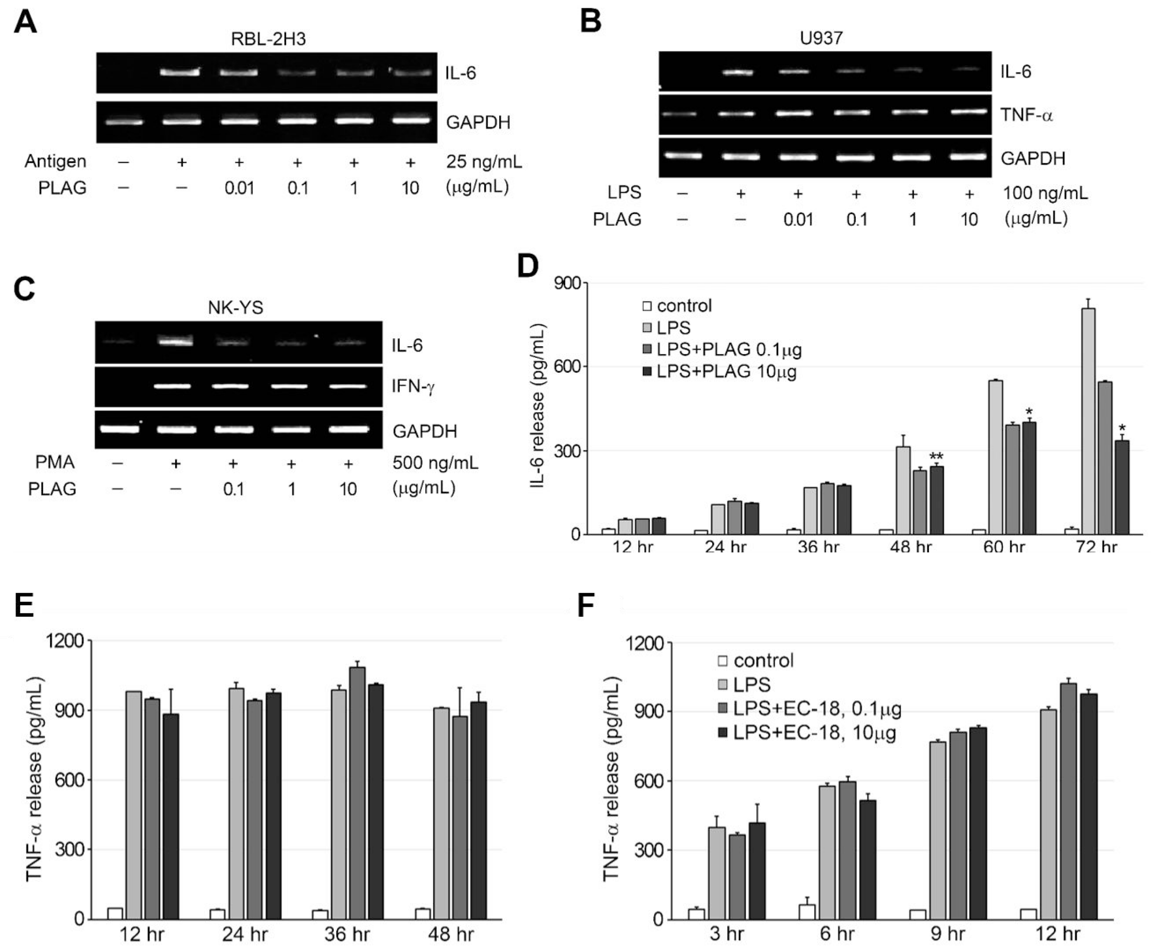

Supplementary Figure 1: PLAG specifically inhibited IL-6 expression with no effect on TNF-α expression. **A**–**C**. The specific inhibition of IL-6 expression by PLAG was confirmed in multiple cell lines by RT-PCR analysis. IL-6 expression in the antigen-stimulated mast cell line RBL-2H3 (A), LPS-stimulated monocytic cell line U937 (B), or PMA-stimulated NK-YS natural killer cells (C) was inhibited by PLAG in a concentration-dependent manner. **D**. THP-1 monocytic cells were treated with 100 ng/mL LPS in the presence or absence of PLAG. The LPS-induced production of IL-6 was inhibited by PLAG. **E**–**F**. RAW264.7 (E) or THP-1 (F) cells were treated with LPS (100 ng/mL) and the indicated concentration of PLAG. Culture medium was harvested at the indicated times and analyzed by ELISA. PLAG treatment had no effect on LPS-induced TNF-α production.

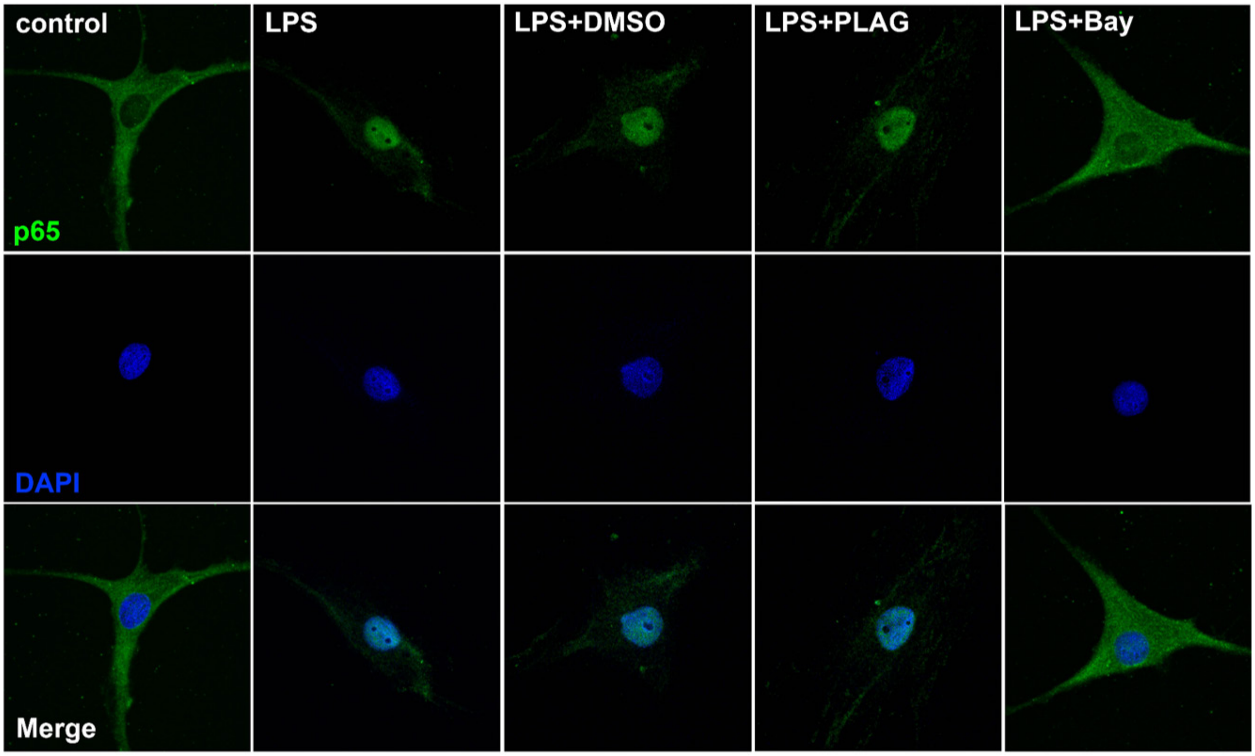

Supplementary Figure 2. Nuclear translocation of activated p65 was not affected by PLAG. LPS treatment of RA-FLSs induced the nuclear translocation of NF-κB subunit p65, which is in the cytoplasm of unstimulated cells. Unlike Bay 11-7082, which showed a strong inhibitory effect on p65 translocation, PLAG exhibited no effect on activation of the NF-κB subunit.
